# Histotripsy: Recent Advances, Clinical Applications, and Future Prospects

**DOI:** 10.3390/cancers17183072

**Published:** 2025-09-19

**Authors:** Mustaqueem Pallumeera, Marcus Hong, Jonathan C Giang, Mina S Makary

**Affiliations:** 1 The Ohio State University College of Medicine, Columbus, OH 43210, USA; mustaqueem.pallumeera@osumc.edu (M.P.); marcus.hong@osumc.edu (M.H.); 2 Northeast Ohio Medical University, Rootstown, OH 44272, USA; jgiang@neomed.edu; 3 Division of Vascular and Interventional Radiology, Department of Radiology, The Ohio State University Wexner Medical Center, Columbus, OH 43210, USA

**Keywords:** histotripsy, radiology, oncology

## Abstract

Histotripsy is a novel, non-invasive ultrasound-based ablative therapy that destroys tissue through focused cavitation. As solid tumors continue to be a major global health burden, there is interest in image-guided ablation techniques that reduce collateral damage and promote immune activation. This narrative review aims to synthesize current advancements, clinical applications, limitations, and future directions of histotripsy in both oncologic and non-oncologic contexts. A comprehensive literature search was conducted from database inception to July 2025. Search terms included combinations of subject headings and keywords such as “histotripsy,” “mechanical ablation,” “ultrasound,” and “solid tumors.” Boolean operators and truncation were used to increase sensitivity. Peer-reviewed studies were included, encompassing preclinical, clinical, and review articles. Reference lists of relevant articles were examined to identify additional sources. Histotripsy has shown strong potential in the treatment of tumors involving the liver, pancreas, kidney, brain, and cardiovascular system. It offers real-time imaging guidance, sharp lesion boundaries, and minimal damage to surrounding structures. Early clinical trials have demonstrated encouraging safety and efficacy, particularly in liver and kidney tumors. Its ability to preserve critical anatomy and stimulate innate and adaptive immune responses through the release of cellular debris and cytokines offers advantages over thermal ablation. Limitations include acoustic aberration, motion-related targeting challenges, and the need for further long-term clinical data. Histotripsy represents a promising advancement in noninvasive tumor ablation. Continued clinical investigation and technological refinement are necessary to validate its therapeutic value and define its role within comprehensive cancer care.

## Simple Summary

Solid tumors remain one of the leading causes of illness and death worldwide, and new treatment methods are needed to improve outcomes while minimizing harm. Histotripsy is an emerging treatment that uses focused sound waves to break down tissue without surgery, heat, or radiation. This technique allows doctors to precisely target tumors while protecting nearby healthy structures through real-time image guidance. It may also help the body’s own immune system to recognize and fight cancer. In this review, we bring together the most up-to-date information on how histotripsy is being studied in different organs, including the liver, kidney, pancreas, brain, and heart. We also discuss the current limitations, such as technical challenges and the need for more long-term patient data. By summarizing the current knowledge and highlighting future directions, this work provides researchers and clinicians with a clearer understanding of the potential role of histotripsy in cancer treatment.

## 1. Introduction

Solid tumors, defined as abnormal masses of tissue without cystic or liquid components, encompass a broad range of malignancies including hepatocellular carcinoma (HCC), pancreatic adenocarcinoma, renal cell carcinoma, and gliomas [1,2,3]. These tumors vary widely in etiology, growth patterns, and response to treatment, necessitating a range of therapeutic approaches tailored to tumor type, stage, and patient-specific factors.

Historically, the cornerstone of solid tumor management has been a combination of surgical resection, systemic chemotherapy, and radiation therapy. While these modalities remain essential, they are often limited by tumor location, comorbidities, or treatment-related toxicity [4,5]. In recent years, there has been interest in less invasive, image-guided approaches that aim to achieve local tumor control with fewer complications and quicker recovery [6]. Among these, percutaneous tumor ablation has emerged as a valuable tool in the treatment of localized disease, offering curative potential for small tumors and meaningful palliation in unresectable cases [7,8,9,10].

Radiofrequency ablation (RFA) and microwave ablation (MWA) rely on thermal energy to generate coagulative necrosis within tumor tissue [5,11,12,13]. Cryoablation uses cycles of rapid freezing and thawing to form intracellular ice crystals, disrupt cell membranes, and may augment antitumor immune responses [7,14]. Irreversible electroporation (IRE), a nonthermal modality, delivers high-voltage electrical pulses that permanently permeabilize cell membranes, enabling tumor destruction while sparing heat-sensitive vascular and biliary structures [4].

The clinical utility of percutaneous ablation is evident when surgery is contraindicated or carries excessive risk. Patients with cirrhosis, impaired performance status, or limited cardiopulmonary reserve may benefit from percutaneous approaches that can be performed under conscious sedation, resulting in reduced hospital stay and lower overall treatment burden (Table 1) [13,15,16]. Even when surgery is indicated, the COLLISION trial demonstrated that oncologic outcomes after ablation were comparable to those after surgery, with decreased adverse events and hospital stay in patients receiving ablation [17].

As global cancer incidence continues to rise, the need for effective, patient-centered, and resource-conscious therapies is growing in parallel. HCC and RCC, in particular, contribute substantially to the worldwide clinical and economic burden due to their increasing prevalence and the complexity of their management [1,3,9]. Image-guided ablation plays a crucial and expanding role in oncologic care, combining precision with accessibility to enhance outcomes across diverse patient populations [18]. Among emerging modalities, histotripsy, a technique that uses focused ultrasound to disrupt tumor tissue mechanically, represents a promising frontier in the evolution of image-guided therapies. Given the novelty of histotripsy, aggregating current findings and exploring future directions is necessary to stay updated on the clinical indications of this emerging technology.

This review aims to provide an overview of the applications of histotripsy in the oncologic treatment paradigm and its impact on reshaping the clinical practice of cancer treatment. It highlights advances in clinical efficacy through a discussion of foundational and up-to-date literature, while also discussing the limitations within each treatment domain.

## 2. Materials and Methods

This review aims to provide a comprehensive overview of histotripsy as an emerging ablation modality in oncology and related fields. The relevant literature was identified through a non-systematic search of PubMed, Scopus, and Google Scholar up to July 2025, using combinations of terms such as “histotripsy,” “noninvasive ablation,” “ultrasound therapy,” “solid tumors,” “immunomodulation,” and specific organ systems including “liver,” “pancreas,” “kidney,” “brain,” and “cardiovascular.” Peer-reviewed original research, preclinical and clinical studies, and review articles were included based on relevance, novelty, and contribution to the understanding of histotripsy’s mechanisms, applications, and limitations.

Priority was given to studies from the past five years and those marking key technological or clinical milestones. Reference list screening was used to identify additional sources. No formal quality appraisal or risk of bias assessment was performed, as the goal was to synthesize current knowledge rather than systematically evaluate treatment efficacy.

## 3. Novelty and Innovation of Histotripsy

The term “histotripsy” is derived from the Greek words “histo,” meaning tissue, and “tripsy,” meaning breakdown [19]. Histotripsy represents a paradigm shift in tissue ablation by leveraging purely mechanical ultrasound-based cavitational mechanisms rather than thermal or ionizing modalities [18]. Tissue destruction is achieved when sufficient negative pressure is applied to a fluid, causing the generation and rapid collapse of cavitation microbubbles at the targeted focal zone [20]. When these bubbles collapse, they generate intense, localized dynamic patterns of oscillation and inertial collapse, creating mechanical forces that mechanically fractionate tissue into subcellular and acellular debris. This process, often termed “mechanical liquefaction,” spares extracellular matrix scaffolding in adjacent zones and preserves vital structures outside the focal region due to sharply demarcated lesion margins. This nonthermal, non-ionizing approach minimizes collateral thermal injury and scarring, providing a favorable safety profile. For instance, preclinical and clinical observations report that ablation zones are absorbed within 1–2 months with minimal fibrosis or damage to adjacent structures [21]. Moreover, histotripsy offers real-time monitoring capability via ultrasound imaging, enabling precise lesion placement and adjustment during treatment. This represents a significant advance over “blind” or less-directly monitored thermal approaches [22].

A comprehensive review by Sandilos et al. explores histotripsy, emphasizing its nonthermal cavitational advantages over traditional thermal methods [23]. Preclinical studies summarized in this review indicate that histotripsy effectively ablates tissue with minimal collateral damage. Furthermore, the #HOPE4LIVER trial reported a 95.5% efficacy rate and a 6.8% complication rate in treating hepatocellular carcinoma and liver metastases using histotripsy [23,24]. This aligns with other early feasibility data demonstrating high technical success in hepatic tumor ablation, accompanied by an acceptable safety profile [25]. The US FDA recently cleared HistoSonics’ Edison system for liver tumor treatment in October 2023, marking its transition from preclinical innovation to clinical reality [26].

Beyond oncologic indications, Sandilos et al. note that histotripsy’s precision and mechanical selectivity suggest promising future applications in various benign and malignant conditions, including benign prostatic hyperplasia, thrombolysis, cardiovascular calcifications, and potentially neuromodulation [23]. Importantly, histotripsy’s ability to generate immunogenic debris and a pro-inflammatory tumor microenvironment positions it at the intersection of ablation and immunotherapy, an emerging frontier in cancer treatment where mechanical ablation may synergize with systemic therapies to reduce recurrence and metastasis [21,22]. Collectively, these attributes, which include mechanical cavitational ablation, real-time imaging guidance, immunomodulatory potential, and broad envisioned applications, highlight the novelty of histotripsy in the landscape of non-invasive therapeutic technologies.

## 4. Mechanisms and Bioeffects of Histotripsy

**Figure 1:**
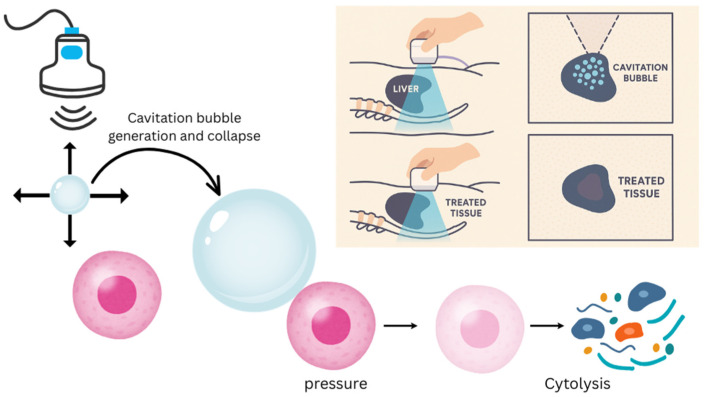
The mechanism of histotripsy centers around the rapid generation and collapse of cavitation bubbles induced by ultrasound waves. When these bubbles rapidly collapse, intense, localized mechanical forces are exerted on targeted tissues and cause cellular destruction.

Key physical parameters govern the efficacy and selectivity of histotripsy. High peak-negative pressures above a cavitation threshold are required to initiate bubble formation. This includes pulse duration, pulse repetition frequency, and focal geometry, which determine bubble dynamics and the extent of mechanical disruption. Real-time ultrasound imaging monitors cavitation activity (“bubble cloud”) and lesion evolution, allowing operators to modulate parameters to ensure complete ablation while avoiding off-target effects (Figure 1) [23]. Bioeffect studies reveal that histotripsy-generated debris is largely acellular, with minimal residual intact tissue. Subsequent clearance by immune cells and remodeling processes leads to the absorption of the ablated region over weeks to months [21].

Expanding on this, Imran et al. characterized the biophysical underpinnings of histotripsy’s immune effects through the phenomenon of “magic bubbles”, which are cavitation bubbles generated during treatment that mechanically disrupt tumor cells and stimulate innate immune pathways. Their study demonstrated that histotripsy enhanced dendritic cell maturation and systemic CD8+ T cell responses, fostering an adaptive antitumor immune response that may synergize with immunotherapies such as checkpoint inhibitors [27]. This mechanistic insight opens avenues for combining histotripsy with immunomodulatory drugs to overcome the tumor’s notoriously suppressive microenvironment.

Cavitational bioeffects also include mechanotransduction leading to cell membrane disruption, vascular fragmentation within the focal zone, and release of intracellular components that can act as damage-associated molecular patterns (DAMPs). These immunogenic signals may recruit and activate antigen-presenting cells, potentiating systemic anti-tumor immunity when used for oncologic indications [21,23]. Histotripsy facilitates the release of DAMPs, pro-inflammatory cytokines, and chemokines to recruit and activate dendritic cells, macrophages, NK cells, and CD8+ T cells. Preclinical models demonstrate effective tumor debulking, reduced metastatic burden, and synergy with checkpoint inhibitors or other immunotherapies [23]. Ongoing studies continue to refine parameter sets for different tissue types and pathologies, aiming to maximize efficacy while preserving surrounding anatomy.

## 5. Liver

### 5.1. Current Applications

Liver neoplasms, the second leading cause of cancer-related deaths globally, account for 850,000 new cases annually [28]. Histotripsy is emerging as an attractive modality for liver tumor ablation, as its unique mechanism has proven especially advantageous in treating tumors located near vascular and biliary structures, where the heat-sink effect limits heat-based approaches.

The precision of histotripsy in the liver was first demonstrated in rodent HCC models, where histotripsy consistently achieved sub-millimeter transition zones and significantly slowed tumor progression relative to controls [29]. Porcine studies comparing histotripsy and microwave ablation showed that while histotripsy created smaller average ablation volumes (4.2 ± 0.5 cm^3^ vs. 6.1 ± 0.4 cm^3^), it preserved surrounding tissue more effectively, particularly near bile ducts and vessels, due to a substantially narrower transition zone (0.2 mm vs. 1.3 mm) [30].

In the THERESA trial, histotripsy was applied to 11 liver tumors across 8 patients, achieving complete technical success and producing median volume reductions of 40% to 70% within three months. Even tumors adjacent to critical hepatic structures were ablated safely, with no procedure-related complications observed [25]. These findings are reinforced by a global safety analysis by Wehrle et al., who reviewed 230 histotripsy procedures involving primary and secondary liver tumors and reported no major device-related complications. Of these 230 procedures, 31 of these tumors were hepatocellular carcinoma. Other treated tumor types included colorectal metastases, neuroendocrine tumors, pancreatic tumors, and breast metastases [31].This broad safety record confirms the reproducibility of histotripsy across various centers and operators, laying the groundwork for standardized clinical deployment.

Clinical trials have established the feasibility and safety of histotripsy across diverse liver tumor contexts. The #HOPE4LIVER trial, a prospective European multicenter study, enrolled 45 patients with 84 liver lesions (47 metastatic and 37 primary). The study demonstrated a technical success rate of 98.8%. By day 30 post-treatment, 90.5% of lesions showed a volume reduction of ≥50%, with over one-third exhibiting more than 75% shrinkage. Importantly, no device-related serious adverse events were reported, highlighting both the efficacy and tolerability of the device in a real-world population [24]. One-year clinical outcomes of this trial for nineteen patients with hepatocellular carcinoma and 28 with metastatic disease demonstrated that, per the primary assessment of nodular or mass-like area of enhancement, the local control rate at 1 year was 63.4%. The post hoc method showed the local control rate at 90%. While there were six serious adverse device-related effects within 30 days, only one non-serious adverse device-related effect was observed after 30 days of treatment. Overall survival rates at one year were 73.3% for patients with HCC and 48.6% for patients with metastatic disease. These results over one year demonstrate persistent efficacy and safety of histotripsy in a real-world environment [32].

Histotripsy’s advantage in treating perivascular tumors is supported by demonstrations in large-animal models that the technique maintains ablative efficacy without damaging adjacent vascular structures [33]. This positions histotripsy as an attractive option for centrally located tumors or those near critical anatomy. In porcine studies, Longo et al. demonstrated that respiratory motion compensation, which modifies the ablation prescription to accommodate ± 1 cm of motion, allowed for 93% completion of the intended ablation volumes [34]. Magnetic resonance-guided histotripsy has also proven effective in delineating cavitation zones, enabling real-time planning and monitoring during treatment [9]. Meanwhile, aberration correction using cavitation emissions has allowed sub-millimeter targeting in heterogeneous tissues such as steatotic liver [35].

### 5.2. Future Directions

Histotripsy represents a promising frontier in the treatment of HCC and liver metastases, with increasing evidence for both local control and systemic immune activation. As clinical data mature, future directions focus on refining precision, integrating immune-based strategies, enhancing device adaptability, and evaluating long-term oncologic outcomes. At the time of publication, the BOOMBOX prospective registry is an ongoing observational study collecting information on the use of the HistoSonics Edison System for treating liver tumors. Its purpose is to understand how different procedural and patient characteristics may affect histotripsy success at 36 h post-procedure (NCT06486454).

The #HOPE4LIVER and THERESA trials have high technical success and favorable safety profiles, supporting expansion into larger, multicenter, randomized trials with survival and recurrence endpoints in the future. Histotripsy’s excellent performance in highly confined ablation zones, with better preservation of adjacent structures such as vasculature and bile ducts, suggests particular value for centrally located or perivascular tumors. Further study is warranted for expanded clinical use. Another consideration is the heterogeneity of tumor environments. Histotripsy is most effective in soft, homogeneous tissue. In cirrhotic livers or tumors with fibrotic or calcified regions, bubble cloud formation and energy deposition can become inconsistent. Knott et al. found reduced effectiveness of histotripsy compared to microwave ablation in fibrotic tissue zones within a porcine model, highlighting the need for tailored treatment algorithms [30].

At the device level, the development of compact, kerf-minimizing phased arrays and 256-element spiral transducers enables more ergonomic and high-resolution delivery of energy through rib spacing, thereby reducing off-target injury and patient discomfort [36,37]. Moreover, AI-assisted aberration correction, utilizing feedback from cavitation emissions or MRI guidance, has demonstrated up to an 80% improvement in focal precision during transcostal liver therapy [35]. While histotripsy represents an advance in tumor ablation, limitations remain in its application to hepatic malignancies. One significant limitation is the treatment of large or deeply seated tumors. Although histotripsy can achieve submillimeter precision in targeting hepatic lesions, the efficacy diminishes with increasing tissue depth due to attenuation and aberration of the ultrasound beam. Advanced targeting systems utilizing phased arrays and aberration correction algorithms have shown promise, but further refinement is needed for reliable delivery across patient anatomies, and clinical translation remains under evaluation [35].

Boiling histotripsy has also demonstrated feasibility in both invasive and transcutaneous liver treatments [38,39]. Similar findings to cavitation histotripsy were reported: complete liquefaction based on histologic analysis, some damage to the body wall, disruption of respiratory motion within the treatment zone, and ultrasound aberrations from the body wall [39]. Additionally, in vivo work on boiling histotripsy to address these aberrations has been successfully conducted [40].

Histotripsy holds further evolving immunomodulatory potential. In murine models, Osada et al. and Qu et al. demonstrated that histotripsy enhanced the infiltration of CD4+ and CD8+ T cells and elevated IFN-γ levels, even inducing abscopal effects when combined with immune checkpoint inhibitors [25,41]. These findings have begun to be translated into human studies, as Vidal-Jove et al. reported systemic regression of extrahepatic lesions and increased serum IFN-γ and TNF-α after liver histotripsy in a metastatic cancer patient [42]. This suggests synergy between histotripsy and immunotherapies, warranting further exploration.

However, for histotripsy to achieve widespread clinical adoption, key areas require exploration. First, while phase I/II studies suggest efficacy, survival outcomes and recurrence rates must be assessed in large-scale randomized trials. Second, synergistic regimens combining histotripsy with checkpoint blockade, kinase inhibitors, or TACE/HAI chemotherapy must be optimized through controlled studies. Third, quantitative tools for real-time treatment monitoring and dose modeling (e.g., acoustic feedback, MRI-based thermometry) are needed to ensure consistency and safety across institutions [33].

## 6. Pancreas

Histotripsy offers distinct advantages over conventional thermal ablation approaches for pancreatic cancer, a tumor type characterized by dense desmoplastic stroma, a highly immunosuppressive microenvironment, and a challenging anatomical location adjacent to critical vascular and biliary structures. Recent studies have explored histotripsy as a treatment option for healthy pancreases and pancreatic tumors in both small and large animal models in vivo [43].

A foundational feasibility study by Gannon et al. employed ultrasound-guided histotripsy in a porcine pancreas model to test the technique’s precision and safety. They successfully created sharply defined ablation zones, averaging 1.5 cm in diameter, as confirmed by imaging and histology, without causing injury to adjacent vital structures, such as the common bile duct or major blood vessels. Importantly, no complications such as pancreatitis or hemorrhage were observed, highlighting histotripsy’s capacity for safe pancreatic tissue ablation even in the presence of complex anatomy and motion [44]. Despite encouraging preclinical results, several limitations currently constrain the clinical application of histotripsy for pancreatic cancer. A major technical limitation is the anatomical complexity of the pancreas, which is surrounded by critical structures. Although Gannon et al. demonstrated targeting with minimal off-target damage in porcine models, this precision depends heavily on real-time imaging and patient-specific planning, which can be difficult to generalize to human subjects with varying pancreatic anatomy [45]. Motion artifacts due to respiration and gastrointestinal peristalsis introduce another obstacle. Unlike liver histotripsy, where respiratory gating strategies have been piloted, the pancreas sits deep within the retroperitoneum and may not be easily stabilized. Gannon et al. noted the difficulty in maintaining acoustic focus throughout a dynamic porcine procedure, and similar challenges are anticipated in humans [44]. These findings were crucial in validating histotripsy as a technically feasible modality for pancreas-targeted interventions, providing the groundwork for clinical translation.

Falk et al.’s synthesis of data from multiple large animal preclinical studies extends to the pancreas, emphasizing the reproducibility of histotripsy in creating uniform tissue fractionation with low complication rates [33]. This characteristic is particularly advantageous in pancreatic tumors, where proximity to major arteries (e.g., the superior mesenteric artery) often limits the efficacy of thermal ablation. However, pancreatic adenocarcinomas are characterized by a highly fibrotic stroma, which may impede bubble formation and reduce the mechanical efficiency of histotripsy [33]. Although histotripsy is nonthermal and spares surrounding structures, its efficacy can be inconsistent in desmoplastic tumors unless cavitation parameters are carefully adjusted [23].

Beyond direct cytoreduction, histotripsy exerts profound immunomodulatory effects in the pancreas. Hendricks-Wenger et al. demonstrated in a murine subcutaneous pancreatic tumor model that histotripsy significantly remodeled the tumor microenvironment. Specifically, treated tumors showed a 2.5-fold increase in CD8+ cytotoxic T lymphocyte infiltration compared to controls, alongside heightened levels of pro-inflammatory cytokines IFN-γ and TNF-α in the tumor milieu, indicative of a shift toward an immune-activated state [45]. These findings suggest that histotripsy induces immunogenic cell death, releasing tumor antigens DAMPs that enhance antigen presentation and T cell priming. Still, the extent to which this translates into meaningful immune priming in human pancreatic cancers remains unclear. Hendricks-Wenger et al. and Imran et al. were able to demonstrate immune activation in murine models, but these findings were based on subcutaneous or orthotopic murine tumors, which lack the full immunosuppressive and fibrotic complexity of human pancreatic adenocarcinoma [27,45].

Osada et al. further highlighted histotripsy’s emerging role as an intratumoral immunotherapy within the pancreas and emphasized the need for further studies to bridge this gap in knowledge. By releasing tumor antigens and DAMPs, histotripsy can transform the immunologically “cold” pancreatic tumor microenvironment into a more immunogenic state, potentially rendering tumors more responsive to immune checkpoint blockade. This rationale supports ongoing efforts to evaluate combination therapies that integrate histotripsy with PD-1/PD-L1 inhibitors and other immunotherapies in both preclinical and clinical settings [46]. Nonetheless, a trial is currently underway evaluating the safety of the HistoSonics Edison System for the treatment of pancreatic adenocarcinomas using histotripsy (NCT06282809).

Technological innovations are critical for the clinical adoption of histotripsy in pancreatic cancer. The presence of gas-filled bowel loops near the pancreas complicates ultrasound propagation, leading to acoustic shadowing and unpredictable cavitation behavior [22]. Advances in phased-array ultrasound transducer technology and real-time cavitation feedback monitoring enable precise targeting of deep-seated pancreatic lesions while minimizing off-target effects. These systems compensate for respiratory motion and acoustic aberrations caused by intervening tissues, enhancing treatment accuracy near vital structures such as the duodenum and central vasculature [22]. Such improvements are essential given the pancreas’s challenging location and the necessity to avoid injury to adjacent tissues.

In a related context, pulsed high-intensity focused ultrasound (HIFU), which shares mechanical ablation principles with histotripsy, significantly enhanced the intratumoral delivery of gemcitabine by 2.7-fold in a murine model of pancreatic cancer. This resulted in improved survival outcomes, suggesting that histotripsy could similarly improve chemotherapeutic penetration and efficacy by disrupting the dense stromal barrier, a key obstacle in pancreatic cancer treatment [47]. Lastly, the lack of validated clinical endpoints limits trial design. Histotripsy is not yet integrated into standard oncologic workflows, and it remains unclear whether ablation-related immune modulation can meaningfully synergize with chemotherapy or checkpoint inhibitors in the pancreas [46,47].

Collectively, these studies highlight the multifaceted therapeutic potential of histotripsy in the treatment of pancreatic cancer. Ongoing research aims to optimize histotripsy therapy methods for targeting the pancreas, to explore the varying thresholds for tissue selectivity, and to develop more accurate computational models that can predict tissue selectivity for specific clinical scenarios [48].

## 7. Kidney

The first use of histotripsy in the kidney demonstrated the feasibility of using transcutaneous focused ultrasound to destroy renal tissue in a normal rabbit. Applications of histotripsy in the kidney are focused particularly on treating primary solid renal tumors such as renal cell carcinoma (RCC), the seventh most common cancer in developed countries and the deadliest urological cancer [49].

Preclinical studies have demonstrated that histotripsy can produce well-demarcated, homogenized ablation zones within the renal parenchyma while preserving adjacent critical structures such as the collecting system, ureter, and renal capsule. Notably, the safety profile remains favorable even in anticoagulated states or when treating central lesions near the urothelium [33,50,51,52,53,54]. Anatomically, the kidney’s complex structure poses challenges, particularly when treating centrally located tumors near the renal pelvis or collecting system. While histotripsy has been shown to avoid thermal damage, studies have demonstrated transient epithelial injury following the treatment of renal pelvis lesions in porcine models, raising concerns about long-term safety in central lesions [54]. Additionally, although histotripsy may be safer than thermal modalities in patients with anticoagulation, the risk of bleeding is not eliminated. Mauch et al. observed minor hematuria and petechiae in anticoagulated pigs, suggesting that the nonthermal mechanism does not entirely prevent hemorrhagic complications [53].

These findings are supported by histologic evidence and advanced imaging techniques such as shear wave elastography [51]. Comparative preclinical studies have demonstrated that histotripsy offers advantages over cryoablation, including larger ablation volumes, reduced perirenal bleeding, and rapid tissue resorption [52]. Additionally, renal function remains preserved post-ablation even when histotripsy is applied near the renal pelvis and proximal ureter, locations typically challenging for conventional ablative therapies [54].

Building on this preclinical foundation, early-phase human trials are now underway. The Clinical Ablation Using Histotripsy for Noninvasive Treatment of Renal Tumors (CAIN, NCT05432232) feasibility trial is evaluating the safety, technical success, and preliminary efficacy of the HistoSonics system in patients with non-metastatic renal tumors ≤ 3 cm [55,56]. Endpoints include complete tumor coverage on imaging and freedom from grade 3 or higher complications, providing the first systematic clinical assessment of histotripsy’s translational potential in urologic oncology.

A second trial, The HistoSonics Edison System for Treatment of Primary Solid Renal Tumors Using Histotripsy (#HOPE4KIDNEY, NCT05820087), is a prospective, multi-center, single-arm pivotal trial designed to evaluate the effectiveness and safety of the HistoSonics Edison System for the destruction of kidney tissue by treating primary solid renal tumors [57]. Data through 90 days for all enrolled subjects will be summarized in a primary analysis, and subjects will be followed for five (5) years post-index procedure, with evaluations at the 14-day, 30-day, 90-day, 180-day, and annual time points. The project commenced in January 2024, with primary completion anticipated by September 2025 and secondary completion expected by July 2030.

Another promising area is the management of centrally located kidney tumors due to histotripsy’s precise mechanical mechanism of action. This is particularly valuable for tumors near or within the collecting system, where thermal ablation poses a higher risk of damaging the urothelium. In porcine models, application of histotripsy to the renal pelvis and proximal ureter resulted in only transient, self-limited urothelial injury, with complete recovery and no long-term impact on renal function [54].

Preclinical studies have also indicated that the mechanical disruption of tumor tissue may lead to the release of tumor-associated antigens, triggering an anti-tumor immune response within the kidney. This immunologic effect is now being studied in combination with immunotherapy, offering a synergistic approach to cancer treatment [22,29,58]. Finally, although there is interest in the immunologic potential of histotripsy, particularly the abscopal effect, most supporting evidence comes from liver or subcutaneous tumor models [28]. Eigner et al. highlighted this gap, noting that while mechanical ablation may enhance antigen presentation, its actual impact on renal tumor immunity has not been demonstrated in humans [58].

Additionally, the integration of artificial intelligence and advanced imaging platforms is enhancing the precision and efficiency of histotripsy procedures. Developments in real-time ultrasound image segmentation and machine learning are enhancing targeting accuracy, facilitating more effective monitoring of the ablation process, and enabling greater procedural automation. Real-time targeting relies on high-quality ultrasound imaging and operator expertise, although developments in machine learning may offer improvements. Miao et al. developed a convolutional neural network to assist in segmenting histotripsy lesions on ultrasound, but this approach has yet to be widely validated [59]. These technologies hold the potential to support personalized ablation strategies and provide intra-procedural feedback, thereby reducing operator dependence and optimizing clinical outcomes [59].

## 8. Brain

Current applications of histotripsy in the brain focus on the ablation of intracranial tissue, with primary research in preclinical models targeting brain tumors, opening the blood–brain barrier (BBB), and neurosurgical ablation. Transcranial histotripsy has been shown to achieve well-demarcated ablation zones in both healthy and tumor-bearing rodent and porcine brains, with MRI and histology confirming tissue homogenization [60,61,62,63].

Histotripsy can be delivered transcranially using MR-guided or ultrasound-guided phased-array systems, with advances in array design and acoustic windowing improving the treatment envelope and targeting accuracy [60,64,65]. A significant obstacle is the acoustic distortion and energy loss caused by the skull, which leads to beam aberration, scattering, and reduced cavitation precision. While phased-array systems and aberration correction algorithms have been developed to compensate for these effects, targeting remains inconsistent, especially in deeper or lateral brain regions [63,65]. Even with MR-guidance and refined focal steering, the transcranial treatment envelope is constrained by skull thickness and heterogeneity [60]. Dosage studies in murine models indicate that lower pulse numbers and multi-point targeting can maximize tumor cell kill while minimizing hemorrhagic complications, with most hemorrhage confined within 1 mm of the ablation boundary [62]. Duclos et al. also found that optimal cavitation thresholds differed between primary and metastatic tumors, highlighting the need for tumor-specific energy parameters [62].

In addition to tumor ablation, histotripsy has been shown to support applications for drug delivery by inducing transient, localized BBB opening at the periphery of the ablation zone with recovery of tight junctions and vascular integrity within weeks [66]. Furthermore, off-target tissue damage remains a concern. Although the blood–brain barrier (BBB) opening induced by histotripsy has been proposed as a therapeutic benefit, Duclos et al. also showed that cavitation can produce variable and uncontrolled BBB disruption depending on pulse settings [66].

Real-time ultrasound and MR imaging are used for treatment monitoring and targeting, with MR thermometry and passive cavitation mapping providing sub-millimeter accuracy [67]. Dual-mode targeting, utilizing magnetic resonance thermometry or acoustic emissions, helps improve focal accuracy. Still, these solutions add complexity and require specialized hardware not yet standard in clinical neurosurgical workflows [59,67].

Histotripsy may have significant use in the dissolution of both intravascular and extravascular clots. Early in vitro experiments have demonstrated its application in addressing intracranial lesions, such as intracerebral hemorrhage and other mass lesions [68]. Moreover, the precision offered by histotripsy, coupled with the acoustic cavitation emission signals that can be used for real-time localization, makes it an attractive option for treating smaller, deeper intracranial targets in various neurological conditions. This includes glioblastoma, Parkinson’s disease, and essential tremor [69].

## 9. Cardiovascular

Applications of histotripsy in the cardiovascular system are primarily in preclinical and early translational research. The most robust application is thrombolysis for the treatment of vascular thrombosis. Histotripsy, including the microtripsy variant, utilizes focused ultrasound to mechanically fragment thrombi, thereby restoring vessel patency without the need for thrombolytic drugs. This approach has demonstrated the ability to recanalize both acute and retracted clots in vitro and animal models, with minimal risk of vessel wall injury and production of clinically insignificant debris particles. Histotripsy can also enhance the efficacy of catheter-directed thrombolytic therapy by increasing thrombus permeability and drug penetration, potentially reducing the required dose of thrombolytics and associated bleeding risk [70,71,72,73]. One key limitation is the difficulty of delivering consistent cavitation in dynamic cardiovascular environments, particularly due to cardiac motion, variable blood flow, and vessel wall compliance. Early work by Maxwell et al. and Zhang et al. established the feasibility of clot fragmentation using controlled cavitation, but these models were mostly static or simplified [70,72]. The presence of pulsatile flow and constantly shifting targets in vivo significantly complicates energy delivery and lesion localization [71].

Another significant application is the creation of intracardiac communications, such as atrial or ventricular septal defects, for the palliation of congenital heart disease. Preclinical studies in neonatal and juvenile animal models have demonstrated that histotripsy can safely and effectively create septal defects through the intact chest, resulting in well-demarcated lesions and favorable intermediate-term outcomes [29,74,75]. However, long-term safety data remain sparse. While Owens et al. demonstrated the functional patency of histotripsy-created defects, no human trials have evaluated histotripsy for congenital interventions, and risks such as off-target embolization or arrhythmia induction remain theoretical but unquantified [75]. In addition, early in vitro and in vivo studies have shown the feasibility of histotripsy guided by 3-dimensional echocardiography for treating mitral valve regurgitation as a non-invasive method of basal chordal cutting [76].

Additional research is exploring histotripsy for cardiac tissue ablation, including arrhythmia substrate modification, with the development of motion correction systems to address cardiac and respiratory motion during treatment [74]. Furthermore, histotripsy is being investigated in hypertrophic cardiomyopathy, particularly in a rat model that reduces wall thickness by 16.2%. This leads to improvements in myocardial strain [77]. At present, these applications remain investigational, with no established clinical guidelines or routine clinical use in the cardiovascular system.

## 10. Limitations

Several challenges surround the utility of histotripsy across clinical scenarios. Histotripsy is currently in the trial phase, with limited availability for most patients [31]. As these trials are being conducted, various limitations are being identified and addressed. Respiratory motion introduces challenges in maintaining treatment accuracy as precise targeting is necessary during specific phases of respiration to avoid collateral injury. Longo et al. addressed this by modifying ablation zone prescriptions to compensate for motion in porcine models [34]. Winterholler et al. demonstrated that utilizing high-frequency jet ventilation resulted in less elongation in the craniocaudal dimension of hepatic treatment zones compared to conventional ventilation. The zone of partially treated tissue resulting from respiratory motion was also found to be narrower [78]. Real-time motion compensation in humans, however, remains technologically intensive and has not yet been widely validated [34].

Vascular thrombosis, particularly portal venous thrombosis, has been observed with histotripsy. The initial clinical proof-of-concept study demonstrated the presence of acute portal vein and hepatic vein thrombi following treatment. All thrombi resolved during a 4-week follow-up period [79]. Similar acute results were obtained in the study that optimized the pulse sequence to minimize damage to the body wall. Thrombi in the primary portal vein, peripheral portal vein, and main hepatic vein were detected [80]. The manufacturer is recommending intraprocedural anticoagulation during histotripsy of central tumors in proximity to the main portal vein branches. However, since no chronic data were reported in this second study, the current findings suggest that the majority of thrombi are non-occlusive or resolve [79].

Treatment size limitations also persist. While the average treated tumor diameter was 1.5 cm in the #HOPE4LIVER trial and 1.4 cm in the THERESA trial, the maximum post-treatment ablation zone reached 3.6 cm across. This established that the device could generate ablation zones large enough to encompass tumors up to approximately 3–4 cm. While multiple overlapping ablations can address larger tumors, this increases procedure time and may compromise uniformity. The THERESA and #HOPE4LIVER trials have demonstrated histotripsy’s safety and efficacy in tumors 3–4 cm, but its scalability for multifocal or diffuse hepatic disease is not yet established [24,25]. Lastly, immunologic effects, though promising, are not fully understood in human liver tumors. While the studies by Worlikar et al. and Osada et al. show enhanced CD8+ T cell activation and dendritic cell priming in murine models, translating these immune responses to humans remains under investigation. Moreover, variability in immune modulation across tumor types could limit the generalizability of these effects [29,46].

Tissue heterogeneity, particularly in the kidney, affects cavitation dynamics as variations in tissue stiffness influence ablation thresholds and lesion uniformity, which complicates dose planning and reproducibility [51]. Qi et al. proposed using dual-frequency pulse schemes to improve focus and lesion sharpness, but these innovations remain experimental [50]. When compared directly with existing treatments, histotripsy lacks long-term outcome data and standardized workflows. Couillard et al. reported similar efficacy between histotripsy and cryoablation in porcine models, but noted longer procedural times and increased variability with histotripsy [52]. Ongoing human trials, such as the CAIN and #HOPE4KIDNEY feasibility study, aim to establish the safety and practicality of histotripsy in treating small renal tumors. However, until the results are published, histotripsy remains an investigational treatment [55].

Another limitation is variability in lesion formation and post-treatment imaging characteristics. Choi et al. reported that histotripsy-treated gliomas in mice showed variable MRI and histologic evolution over time, with some lesions exhibiting incomplete liquefaction or peripheral edema [61]. Hardware limitations also persist. Ruger et al. proposed a polyolefin-based cranioplasty device to facilitate more efficient ultrasound transmission. However, this approach is only applicable in post-craniotomy settings and does not address intact skull treatments [64]. Landry and Brown showed that even minor adjustments in pulse duration and frequency significantly altered lesion size and shape in rat brains, indicating a narrow therapeutic window [81]. Finally, while histotripsy holds potential for the treatment of deep-seated brain tumors, its translation into human use is hampered by a lack of survival model data, limited large-animal testing, and the absence of clinical trials [60]. As highlighted by Worlikar et al., rigorous safety profiling and motion compensation strategies must precede any attempts at clinical deployment [29]. 

The presence of pulsatile flow and constantly shifting targets in vivo in vascular regions significantly complicates energy delivery and lesion localization [71]. To address this, Miller et al. developed a motion-tracking cardiac histotripsy system. However, real-time correction remains computationally and technically intensive [74]. Finally, vascular safety and endothelial integrity are under-characterized. While the nonthermal mechanism avoids heating, the mechanical energy can still injure the endothelium or dislodge plaque, particularly in calcified or diseased vessels [29]. 

## 11. Conclusions

Histotripsy is a novel, noninvasive, and nonthermal ablation modality that offers precise tissue targeting with minimal collateral damage. Its cavitational mechanism is well-suited for tumors near critical structures, with encouraging preclinical and early clinical data across hepatic, renal, pancreatic, and intracranial applications. Emerging evidence also suggests potential synergy with immunotherapies.

Despite this promise, histotripsy faces challenges, including targeting in complex anatomy, limited reproducibility, and an incomplete understanding of its immunologic effects in humans. Most data remain preclinical or early phase, and questions persist regarding long-term outcomes and optimal clinical integration. Continued translational research and large-scale trials are crucial for defining histotripsy’s role in multimodal therapy and determining its potential as a cornerstone of non-invasive cancer treatment.

## Figures and Tables

**Table 1:** An overview of ablative techniques.

Technique	Mechanism	Key Features	Limitations
Histotripsy	Nonthermal mechanical tissue fractionation using focused ultrasound-induced cavitation	Noninvasive, precise targeting, minimal collateral damage, real-time imaging, mechanical and immunological efficacy	Emerging clinical data, targeting challenges in mobile anatomy, limited immunologic understanding, limited indications currently
Radiofrequency Ablation (RFA)	Thermal injury via high-frequency alternating current	Widely used, effective for small tumors, percutaneous or laparoscopic access	Heat-sink effect, limited precision, risk of thermal damage to adjacent structures
Microwave Ablation (MWA)	Dielectric heating through microwave energy	Faster and larger ablation zones than RFA, less susceptible to heat sink	Collateral thermal injury, limited use near heat-sensitive structures
High intensity Focused Ultrasound (HIFU)	Thermal ablation via focused ultrasound heating	Noninvasive, MRI-guided, precise heating, acoustic cavitation	Thermal latency, bone interference, skin burns, long treatment time
Cryoablation	Cell death via freeze–thaw cycles using cryoprobes	Visible ice-ball on imaging, less pain, potential immune activation	Longer procedure time, risk of hemorrhage, ice-ball unpredictability
Irreversible Electroporation (IRE)	Nonthermal cell death by high-voltage electric pulses disrupting membranes	Spares extracellular matrix, useful near vessels and ducts	Requires general anesthesia, cardiac synchronization, limited availability

## Abbreviations

The following abbreviations are used in this manuscript:

HCC	Hepatocellular Carcinoma
RFA	Radiofrequency Ablation
MWA	Microwave Ablation
IRE	Irreversible Electroporation
DAMPs	Damage Associated Molecular Patterns
HIFU	High-Intensity Focused Ultrasound
RCC	Renal Cell Carcinoma
CAIN	The Clinical Ablation Using Histotripsy for Noninvasive Treatment of Renal Tumors
BBB	Blood–Brain Barrier
